# Combined Transcranial Direct Current Stimulation and Transcutaneous Cervical Vagus Nerve Stimulation for Anxiety and Depression: An Exploratory Pilot Study

**DOI:** 10.3390/brainsci16101004

**Published:** 2026-09-22

**Authors:** Maria Marchiș, Magdalena Iorga, Iustina-Gabriela Mihăianu, Maria-Manuela Apostol

**Affiliations:** 1 Faculty of Psychology and Educational Sciences, “Alexandru Ioan Cuza” University, 700554 Iasi, Romania; maria.marchis@student.uaic.ro (M.M.); maria_manuela.apostol@umfiasi.ro (M.-M.A.); 2 Faculty of Medicine, “Grigore T. Popa” University of Medicine and Pharmacy, 700115 Iasi, Romania; nutr-rom-514@students.umfiasi.ro; 3 Prolife Clinics, 700020 Iasi, Romania

**Keywords:** transcranial direct current stimulation, transcutaneous cervical vagus nerve stimulation, anxiety, depression, neuromodulation

## Abstract

**Highlights:**

**What are the main findings?**

- Participants experienced a 52.0% decrease in anxiety (BAI scores) and a 55.9% decrease in depression (BDI scores) after 10 sessions, with depression remission rates jumping from 8.3% to 83.3%.
- The combined tDCS and tcVNS protocol was feasible and well-tolerated with no reported adverse events, while older participants with more severe baseline anxiety and depression demonstrated the highest response rate.

**What are the implications of the main findings?**

- Combining non-invasive neuromodulation techniques may offer a highly effective, safe and accessible alternative to traditional psychiatric treatments.
- The distinct responder profile implies that future clinical protocols can be more optimized and personalized for older populations or individuals presenting more severe, comorbid baseline symptoms.

**Abstract:**

Anxiety and depressive disorders are among the leading causes of disability worldwide, yet current treatments remain limited by modest efficacy and restricted accessibility; non-invasive neuromodulation techniques such as transcranial direct current stimulation (tDCS) and transcutaneous cervical vagus nerve stimulation (tcVNS) have emerged as promising alternatives whose combination may produce complementary clinical effects. **Methods:** This proof-of-concept pilot study evaluated the feasibility, tolerability, and preliminary clinical effects of a multimodal three-component protocol comprising tDCS and tcVNS delivered alongside supportive psychological counseling in 12 adults (Mage = 54.75 ± 17.47 years) with clinically significant anxiety and depressive symptoms. Participants received 10 consecutive sessions of anodal tDCS over the left dorsolateral prefrontal cortex (2 mA, 30 min) followed by tcVNS with PEMF, with symptoms assessed using the Beck Anxiety Inventory (BAI) and Beck Depression Inventory (BDI) and pre–post comparisons performed via the Wilcoxon signed-rank test. **Results:** Significant reductions were observed for both outcomes: BAI scores decreased by 52.0% (Z = −3.059, p_exact_ < 0.001, p_asymp_ = 0.002, r = 0.883) and BDI scores by 55.9% (Z = −3.059, p_exact_ < 0.001, p_asymp_ = 0.002, r = 0.883); the proportion of participants in the minimal BDI-II symptom range rose from 8.3% to 83.3%, and no adverse events were reported. Standard clinical response rates (≥50% score reduction from baseline) were achieved by 50.0% of participants for depression (BDI) and 41.7% for anxiety (BAI), with 25.0% demonstrating a concurrent dual response on both measures. Exploratory profiling and multivariable continuous modeling indicated that higher cumulative improvement was descriptively observed in older participants with greater baseline symptom severity, with baseline anxiety showing the strongest continuous standardized association (β = 0.548, *p* = 0.104). These are hypothesis-generating observations only. **Conclusions:** The combined three-component package (tDCS, tcVNS, and concurrent supportive counseling) appears feasible, well tolerated, and associated with pre–post symptom reductions that, in the absence of a control condition, cannot be causally attributed to the intervention, warranting further investigation in adequately powered randomized sham-controlled trials.

## 1. Introduction

Anxiety and depressive disorders represent one of the most significant global public health challenges, as Global Burden of Disease estimates for 2021 indicated that more than 359 million people were affected by anxiety disorders and approximately 332 million were living with a depressive disorder; these figures are projected to exceed 500 and 460 million, respectively, by 2040 [1]. The COVID-19 pandemic accelerated this trend, with approximately 76.2 million new cases of anxiety disorders (+25.6%) and 53.2 million new cases of major depression (+27.6%) recorded in 2020 alone relative to pre-pandemic estimates [2].

The economic burden is substantial, as depression and anxiety are estimated to cost approximately 12 billion working days annually, equivalent to approximately US 1 trillion dollars in lost productivity [3]. These figures carry additional clinical relevance given a frequently underestimated aspect of the two conditions: their co-occurrence.

In clinical samples, the vast majority of patients with depression simultaneously meet the criteria for a diagnosable anxiety disorder, which justifies the concurrent assessment of both symptom dimensions in therapeutic and research contexts [4].

Despite therapeutic progress, the clinical benefit of antidepressant pharmacotherapy remains modest, and large-scale real-world data suggest that complete remission is achieved in fewer than 35% of treated patients, substantially below earlier estimates of 67% [5,6]. The limited efficacy of first-line interventions, combined with delayed onset of action, patient non-adherence due to adverse effects, and restricted access to mental health services in many regions, has driven increasing interest in complementary, non-invasive, well-tolerated approaches.

### 1.1. Transcranial Direct Current Stimulation

Non-invasive neuromodulation has established itself over the past decade as one such direction, among which transcranial direct current stimulation (tDCS) modulates cortical excitability through subthreshold polarization of the neuronal membrane potential: anodal stimulation depolarizes neurons and increases their excitability, while cathodal stimulation produces the opposite, hyperpolarizing effect, and in the context of depression and anxiety, the canonical anodal target is the left dorsolateral prefrontal cortex (DLPFC), a region characterized by relative hypoactivity during depressive episodes and central to top-down control over limbic circuits, particularly amygdala reactivity. A meta-analysis encompassing 56 studies and 2349 participants confirmed moderate efficacy of 2 mA tDCS applied over the left DLPFC in reducing depressive symptomatology and further identified this region as a transdiagnostic common mechanism relevant to both anxiety and depression [7]. The relevance of a shared prefrontal target is particularly notable given the frequent co-occurrence of anxiety and depression; consequently, interventions capable of concurrently addressing both clinical dimensions offer distinct therapeutic advantages. Beyond its effects on cortical excitability, tDCS has demonstrated beneficial effects across a broad clinical spectrum, including psychiatric conditions, most notably depression and schizophrenia, as well as neurological disorders, and it has been applied in the rehabilitation of cognitive, motor, and sensory functions following stroke. The durability of these effects is thought to be mediated by bidirectional modifications in postsynaptic connectivity, analogous to long-term potentiation and long-term depression, both of which operate through NMDA receptor-dependent mechanisms. Given that pathological alterations in neuroplasticity constitute a core feature of psychiatric disorders, tDCS represents a particularly compelling therapeutic avenue: rather than merely compensating for a neurochemical deficit, it acts directly on the maladaptive plasticity that sustains the disorder, offering the possibility of more durable neurobiological change.

tDCS modulates neuronal excitability in the prefrontal cortex, and this exerts indirect “top-down” control on the nervous system. Combining tDCS with psychotherapy amplifies these effects: by restoring top-down prefrontal control, tDCS may function as a biological accelerator of the therapeutic process, reducing compulsive negative rumination and facilitating cognitive change.

A key advantage of tDCS over other biological interventions is its well-documented safety profile, as reported adverse effects are mild and transient, with the most common being tingling, itching, local erythema, mild headache, and discomfort at the electrode site, with occurrence rates that do not differ significantly from sham stimulation in controlled studies (tingling: 39.3% active vs. 32.9% sham; headache: 14.8% vs. 16.2%) [8]. Serious adverse events have not been documented in the literature under conventional protocols, and a systematic review specifically dedicated to repeated tDCS sessions confirmed that repeated application does not increase risk compared to sham, that the incidence of adverse effects does not accumulate with total stimulation dose, and that no diagnostic group has shown particular vulnerability [9]. The international safety consensus, developed under the auspices of the ESBS and IFCN, concludes that conventional protocols (≤4 mA, ≤40 min, electrodes ≥25 cm^2^) fall well below the tissue injury threshold demonstrated experimentally, and that their use in clinical and research settings is justified and free of significant risk [10,11].

Nevertheless, tDCS monotherapy presents limitations, as the multicenter DepressionDC trial found no significant difference between active tDCS and sham stimulation when added to selective serotonin reuptake inhibitor (SSRI) treatment: MADRS improvements at six weeks were −8.2 versus −8.0 for active and sham conditions, respectively [12]. This finding suggests that isolated cortical stimulation may be insufficient to engage the full prefrontal–limbic network implicated in affective pathology and raises the question of whether a mechanistically complementary second modulation pathway might overcome this ceiling effect. If the limits of cortical monotherapy argue for a second pathway, the vagus nerve provides an attractive candidate, one that acts bottom-up from the periphery rather than top-down from the cortex, and targets partly overlapping but distinct circuits [13].

### 1.2. Transcutaneous Cervical Vagus Nerve Stimulation

The vagus nerve is the primary pathway through which the parasympathetic nervous system communicates with internal organs, transmitting information bidirectionally between brain and body, and approximately 80% of its fibers are afferent, carrying ascending signals [14]. This architecture explains why vagal stimulation can influence mood and anxiety. When the organism perceives threat, the sympathetic nervous system mobilizes an alarm response, increased heart rate, accelerated respiration, muscle tension, and heightened alertness. This response becomes pathological when chronically sustained, as occurs in anxiety and depressive disorders.

The vagus nerve is the principal counterbalance to this state; through its parasympathetic activity, it is responsible for returning the organism to a calm state by slowing down heart rate, supporting digestion, and facilitating recovery. Activation of vagal afferents produces, via baroreflex mechanisms, both parasympathetic stimulation and sympathetic inhibition [15]. High vagal tone is associated with better emotional regulation and faster stress recovery, whereas low vagal tone is consistently observed in depression and anxiety, a pattern that has led to heart rate variability (HRV) being proposed as a physiological marker of adaptive flexibility.

Vagal stimulation reinforces this rebalancing mechanism as ascending vagal signals reach brain regions that regulate mood, arousal, and stress reactivity, specifically the locus coeruleus (noradrenaline), the raphe nuclei (serotonin), and the limbic system, the same circuits targeted by antidepressant pharmacotherapy [16,17]. An additional mechanism of relevance to depression involves the “inflammatory reflex” vagal activation, which reduces the release of pro-inflammatory cytokines, and a significant proportion of depressed patients exhibit low-grade persistent inflammation with elevated inflammatory markers [15,18]. Vagal stimulation thus acts at two levels simultaneously: it modulates the autonomic alarm response and tempers the inflammatory substrate that sustains it. Stimulation of vagal afferents also promotes neuroplasticity through BDNF release [19].

Transcutaneous cervical vagus nerve stimulation (tcVNS) targets the vagal trunk directly at the neck, a point of greater anatomical accessibility than the auricular branch. Although the cervical approach has been less studied than the auricular variant (taVNS), the available evidence in a double-blind, randomized, sham-controlled trial in patients with post-traumatic stress disorder was promising; tcVNS significantly reduced sympathetic responses to stress and attenuated associated inflammatory activation [20].

### 1.3. Rationale and Aims

The present study stems from the observation that tDCS and tcVNS act through mechanistically complementary pathways: tDCS provides cortical top-down modulation of prefrontal executive control, while vagal stimulation acts bottom-up through subcortical monoaminergic and autonomic circuits. Importantly, although the auricular variant of transcutaneous vagal stimulation (taVNS) has accumulated a growing clinical evidence base for depression and anxiety, the cervical approach in combination with tDCS has not, to our knowledge, been evaluated in a dedicated clinical framework for affective disorders [21,22]. It should be noted that the tcVNS device used in this study operates on a pulsed electromagnetic field (PEMF) principle rather than direct electrical stimulation of the vagal trunk, and the rationale for its inclusion rests on its documented effects on autonomic regulation and vagal tone modulation via non-invasive cervical application.

The present research was designed as a proof-of-concept feasibility pilot study. The primary objective was to evaluate the feasibility and tolerability of a combined tDCS + tcVNS protocol administered over ten consecutive sessions, with secondary evaluation of clinical effects on anxiety (BAI) and depression (BDI). Because we aimed to evaluate a protocol study, the following hypotheses have been considered.

**H1.**  *A 10-session combined protocol will be feasible to administer and well tolerated by adults with anxiety and depressive disorders, demonstrating high compliance and no serious adverse effects*.

**H2.**  *Sequential dual-target neuromodulation of central pathways through tDCS and peripheral pathways through tcVNS will result in statistically significant reductions in self-reported anxiety symptoms (as measured by the BAI) and depressive symptoms (as measured by the BDI) from baseline to post-intervention*.

**H3.**  *The combined effects of prefrontal cortical stimulation and vagal neuromodulation will produce high clinical remission rates for depressive symptoms, with a majority of participants expecting to shift from clinically significant baseline scores to subclinical thresholds after intervention*.

**H4.**  *The clinical efficacy of combined top-down tDCS and bottom-up tcVNS neuromodulatory mechanisms will be influenced by baseline symptom severity and age, producing greater symptom reduction and higher remission rates in older adults with severe baseline anxiety and depression symptoms than in younger patients with moderate baseline severity*.

## 2. Materials and Methods

### 2.1. Participants

A one-group pretest–post-test design was employed. Twelve participants (n = 12) were consecutively enrolled. All participants completed all ten sessions with no interruptions or protocol deviations. No adverse events were reported during the study.

Inclusion criteria were clinically significant anxiety and/or depressive symptomatology confirmed by psychological evaluation; age ≥ 18 years and resistance or lack of progress with pharmacotherapy. Clinically significant symptomatology was confirmed through structured psychological evaluation conducted by a licensed clinical psychologist prior to enrolment. Formal DSM-5 diagnostic interviews were not administered as part of this feasibility protocol.

Exclusion criteria comprised psychiatric contraindications (active psychotic episodes, diagnosis of epilepsy); cardiovascular contraindications (cardiac pacemakers, severe arrhythmias, bradycardia); neurological contraindications (intracranial aneurysm, metallic implants, cochlear implant); dermatological contraindications (skin damage, dermatitis, open wounds, infections, or scars at the cervical electrode site); and other absolute contraindications to transcranial neuromodulation.

All participants received written and verbal information concerning the intervention protocol, associated risks, data collection procedures, and confidentiality protections. No incentives were offered for participation. Participants were informed of potential side effects, including tingling, itching, redness, or pressure sensitivity. Written informed consent was obtained prior to the commencement of any intervention.

### 2.2. Procedure

The study was conducted between April and July 2026. All therapeutic procedures were conducted by a clinical psychologist certified as a TES (transcranial electrical stimulation) practitioner. Each session followed a fixed sequence: tDCS was administered first, immediately followed by tcVNS with PEMF, with supportive psychological counseling delivered concurrently throughout both neuromodulation components. Participants receiving pharmacotherapy continued their existing regimen unchanged throughout the study period; no medication adjustments were made as part of the protocol. Adverse events were monitored at each session through structured verbal inquiry; participants were asked to report any discomfort, tingling, headache, or skin irritation. No adverse events were reported during the study.

Each participant received a protocol consisting of 10 consecutive daily sessions comprising three concurrent or sequential components:

(1) Transcranial direct current stimulation (tDCS). Anodal stimulation was applied over the left dorsolateral prefrontal cortex (F3) and cathodal stimulation over the right dorsolateral prefrontal cortex (F4), using the international 10–20 EEG system, at an intensity of 2 mA for 30 min per session. Electrode placement targeted prefrontal regions involved in emotional regulation, in accordance with validated protocols for depression and anxiety [23,24].
(2) Transcutaneous cervical vagus nerve stimulation (tcVNS). Non-invasive vagus nerve stimulation was performed using a pulsed electromagnetic field (PEMF)-based device. The device delivers low-intensity, pulsed magnetic fields applied externally around the cervical region to stimulate the vagus nerve without the use of electrical currents or conductive gels. The stimulation protocol employed a frequency of 32 Hz, selected according to the device-specific protocol for stress and anxiety reduction. Each PEMF stimulation session lasted for 30 min. During the intervention, the device was positioned around the neck to allow localized electromagnetic stimulation of the cervical vagal region. The cervical approach was selected for its direct access to the vagal trunk and its documented effects on vagal tone modulation, sympathetic reactivity reduction, neurogenic inflammation, and limbic circuits implicated in anxiety and depression [16,17,20].
(3) Supportive psychological counseling. Individual sessions of psychological counseling were conducted concurrently with neuromodulation—the intervention included emotional support, active listening, psychoeducation, and facilitation of adaptive coping strategies. Because counseling was not standardized across participants and no comparison group received neuromodulation without counseling, its independent contribution to outcomes cannot be separated from the neuromodulation effects; the results therefore reflect the effect of the multimodal package.

All 12 enrolled participants completed all 10 scheduled sessions without any interruptions or protocol deviations.

### 2.3. Outcome Measures

Beck Anxiety Inventory [25]. A 21-item self-report instrument with scores ranging from 0 to 63, administered using the validated Romanian adaptation [26]. Severity thresholds: 0–7 minimal, 8–15 mild, 16–25 moderate, and ≥26 severe. The BAI demonstrates strong psychometric properties [27].

Beck Depression Inventory [28]. A 21-item self-report instrument with scores ranging from 0 to 63, administered using the validated Romanian adaptation [29]. Severity thresholds: 0–13 minimal symptom range, 14–19 mild, 20–28 moderate, and ≥29 severe (Cronbach’s α = 0.86 in clinical samples).

### 2.4. Statistical Analysis

Normality of continuous distributions was assessed with the Shapiro–Wilk test (recommended for N < 50) [30] in conjunction with the formal inspection of distributional morphology via skewness and kurtosis indices alongside their corresponding standard errors (SE). To enhance descriptive and inferential precision, point estimates across baseline, post-intervention, and delta change scores are reported with two-tailed 95% confidence intervals (95% CI). Given the non-normal distribution of Δ BAI (W = 0.799, *p* = 0.009) and the small sample size, pre–post comparisons were conducted with the Wilcoxon signed-rank test [31]. Given the small sample size (n = 12), both exact two-tailed significance values (p_exact_) and asymptotic values (p_asymp_) were computed. Effect size was quantified using two complementary indices: r = |Z|/√N and Cohen’s d on paired differences [32].

To evaluate multidimensional treatment responsiveness, individual percentage score reductions from baseline were calculated for anxiety (% BAI = (BAI pre − BAI post)/BAI pre * 100) and depression (% BDI = (BDI pre − BDI post)/BDI pre * 100). A cumulative clinical improvement score was defined as the total sum of absolute point reductions across both inventories (ΔTotal = |ΔBAI| + |ΔBDI|). A standard clinical response was defined as a ≥50% reduction from baseline score, and a concurrent dual response was defined as meeting this criterion on both the BAI and BDI simultaneously.

To explore candidate clinical correlates of response, participants were categorized into two distinct outcome profiles: Moderate Responders (ΔTotal ≤ 30 points) and High Responders (ΔTotal > 30 points). Because this feasibility pilot was not preregistered, this threshold was established post hoc based on sample distribution characteristics (approximating the sample mean of M = 29.75 and the 75th percentile of Q3 = 33.00), capturing a natural bimodal separation in clinical gains strictly as an exploratory descriptive heuristic for data visualization rather than an a priori confirmatory clinical benchmark.

To evaluate baseline characteristics continuously across the entire cohort (n = 12) without the statistical pitfalls of artificial dichotomization, multivariable linear regression was performed with age, baseline BAI, and baseline BDI simultaneously predicting cumulative improvement (ΔTotal). Given the uncontrolled pilot design, regression parameters and correlation coefficients are interpreted strictly as exploratory associations and hypothesis-generating covariations rather than directional causal predictors or mediational effects. Non-parametric Mann–Whitney U tests were retained exclusively for exploratory comparisons across gender subgroups on percentage score reductions and cumulative improvement.

Bivariate associations across all continuous demographic, baseline, and change indices were examined using a full Spearman rank-order correlation matrix. To prevent mathematical redundancy and minimize multiple testing burden, derived percentage score reductions were excluded from the correlation matrix, restricting the analysis to eight core continuous variables. Statistical significance was set at α = 0.05 (two-tailed). Given the exploratory pilot design (n = 12), significance values are reported without family-wise multiplicity adjustment (p_uncorrected_) and are interpreted strictly as hypothesis-generating trends rather than confirmatory inferences.

Analyses were performed using IBM SPSS Statistics (Version 26.0). No correction for multiple comparisons was applied, consistent with the exploratory nature of the study.

### 2.5. Ethical Approval

The research was conducted in accordance with the Declaration of Helsinki and approved by the Ethical Research Committee of the Doctoral School, Faculty of Psychology and Educational Sciences, “Alexandru Ioan Cuza” University Iasi, Romania (approval No. 511/3 April 2024) and by the Ethical Research Committee of Prolife Clinics, Iasi, Romania (approval No. 405/22 July 2026).

## 3. Results

### 3.1. Sample Characteristics

The sample comprised n = 12 participants (age M = 54.75 years, SD = 17.47, Mdn = 61.0, range = 22–75), predominantly women (n = 10, 83.3%). Initial BAI scores (M = 33.17, SD = 8.47) and BDI scores (M = 22.33, SD = 6.07) situated the sample, on average, in the severe and moderate symptomatology zones, respectively. Individual data are presented in Table 1.

### 3.2. Normality

The Shapiro–Wilk test (Table 2) confirmed normal distributions for most variables (*p* > 0.05), except for ΔBAI (W = 0.799, *p* = 0.009). Distributional shape analysis confirmed that Δ BAI displayed substantial negative skewness (skewness = −1.179, SE = 0.637) and near-zero kurtosis (kurtosis = −0.011, SE = 1.232). This negative skewness was clinically driven by high-responding participants exhibiting extensive symptom clearance (raw point drops ≥ 31), thereby shifting the lower tail of the distribution. Conversely, depressive symptom changes (ΔBDI) exhibited symmetrical, mesokurtic properties (skewness = −0.383, SE = 0.637; kurtosis = −0.804, SE = 1.232; W = 0.925, *p* = 0.329). Point estimates, distributional parameters, and 95% confidence intervals across all primary variables are summarized in Table 2. This divergence confirmed the statistical necessity of non-parametric modeling for primary outcome evaluations.

### 3.3. Pre–Post Effects of the Combined tDCS + tcVNS Intervention

The Wilcoxon signed-rank test revealed statistically significant reductions for both anxiety and depression (Table 3).

BAI scores decreased from M = 33.17 (SD = 8.47) to M = 15.92 (SD = 9.73), a 52.0% reduction (T = 0, Z = −3.059, p_exact_ < 0.001, p_asymp_ = 0.002, r = 0.883).

BDI scores decreased from M = 22.33 (SD = 6.07) to M = 9.83 (SD = 5.56), a 55.9% reduction (T = 0, Z = −3.059, p_exact_ < 0.001, p_asymp_ = 0.002, r = 0.883).

Value T = 0 confirms that all 12 participants showed improvement in both measures following the intervention.

Standard clinical response rates (≥50% score reduction from baseline) were achieved by 50.0% (n = 6) of participants for depression (BDI) and 41.7% (n = 5) for anxiety (BAI), with 25.0% (n = 3) demonstrating concurrent dual response on both measures.

### 3.4. Effect Sizes

Both effect size indices (Table 4) indicated large-magnitude effects. The Wilcoxon r coefficient (0.883) substantially exceeds the conventional large-effect threshold (r ≥ 0.50). Cohen’s d values on paired differences (BAI: d = −1.667; BDI: d = −2.107) reached the level of very large effects (d ≥ 0.80), indicating practical and clinical relevance that exceeds what is typically reported in neuromodulation studies with comparable designs [33].

### 3.5. Clinical Severity Category Shifts

Analysis of clinical categories (Table 5) revealed important shifts in severity profile. Prior to the intervention, 83.3% (n = 10) of the participants met the criterion for severe anxiety (BAI ≥ 26), and no participant was in the minimal category.

Post-intervention, the proportion with severe anxiety decreased to 8.3% (n = 1), and 25.0% (n = 3) reached the minimal level. For depression, the proportion scoring within the minimal symptom range on the BDI (scores 0–13) increased from 8.3% (n = 1) to 83.3% (n = 10), noting that one participant (P07) was already within this minimal symptom range at baseline (score = 11) and demonstrated a further score reduction post-intervention (score = 6).

### 3.6. Correlational Analyses

The Spearman correlation matrix (Table 6) revealed several significant associations. Age correlated positively with pre-intervention BDI (ρ = 0.60, p_uncorrected_ = 0.040), indicating that older participants presented with more severe depressive symptoms at baseline.

Pre-intervention BAI correlated negatively with pre-intervention BDI (ρ = −0.63, p_uncorrected_ = 0.027), indicating that participants presenting with higher baseline anxiety reported comparatively lower baseline depressive symptom severity within this pilot cohort.

Pre-intervention BAI correlated negatively with ΔBAI (ρ = −0.58, p_uncorrected_ = 0.050), suggesting that participants with more severe anxiety at baseline showed larger reductions.

Post-intervention BAI correlated positively with ΔBDI (ρ = 0.73, p_uncorrected_ = 0.007), suggesting that residual anxiety level was exploratorily correlated with the magnitude of depression improvement.

Given the exploratory pilot scope and the absence of multiplicity adjustment across these 28 pairwise comparisons, these associations are interpreted strictly as provisional observational trends.

### 3.7. Patient Response Profiles

Stratification of participants by cumulative improvement (ΔTotal > 30 points) identified two response profiles: Moderate Responders (n = 9, 75.0%) and High Responders (n = 3, 25.0%).

Descriptively, the three individuals comprising the High Responder tier (P01, P06, P08) presented with an older age distribution (discrete values: 52, 68, and 70 years; median = 68.0) and elevated baseline symptom severity (individual BAI scores: 41, 35, and 37; individual BDI scores: 21, 31, and 22) relative to Moderate Responders (Table 7, Figure 1).

The baseline demographic and clinical characteristics stratified by treatment response profile are presented in Figure 1.

To evaluate candidate baseline characteristics continuously without artificial dichotomization, and to avoid embedding inferential statistics in descriptive text, an exploratory multivariable linear regression model was performed across the unsegmented cohort (n = 12) and is reported in a dedicated table (Table 8). In multivariable linear regression modeling across the entire unsegmented cohort (n = 12), baseline age, baseline BAI, and baseline BDI accounted for 38.9% of the variance in cumulative symptom improvement (R = 0.624, R^2^ = 0.389, adjusted R^2^ = 0.160, F(3, 8) = 1.700, *p* = 0.244). As presented in Table 8, baseline anxiety exhibited the largest standardized coefficient (B = 0.997, SE = 0.544, β = 0.548, t = 1.834, *p* = 0.104), followed by age (B = 0.313, SE = 0.313, β = 0.355, t = 1.000, *p* = 0.347) and baseline depression (B = 0.718, SE = 0.887, β = 0.282, t = 0.810, *p* = 0.441). Although formal statistical significance was constrained by the pilot sample size, the directionality and magnitude of these continuous parameters converge with the descriptive stratification, providing preliminary support for the hypothesis that older individuals with elevated baseline severity may experience heightened clinical responsiveness.

Mann–Whitney U comparisons indicated no statistically significant differences between male (n = 2) and female (n = 10) participants across percentage BAI reduction (U = 6.000, *p* = 0.389), percentage BDI reduction (U = 7.000, *p* = 0.519), or total point drop (U = 8.500, *p* = 0.747) (Table 9).

## 4. Discussion

### 4.1. Feasibility and Clinical Trajectories

Before interpreting these findings, a fundamental design constraint must be acknowledged. The absence of a control arm, blinding, and standardized counseling means that observed pre–post changes cannot be causally attributed to the neuromodulation protocol; spontaneous fluctuation, regression to the mean, and non-specific effects remain viable alternative explanations. The present proof-of-concept feasibility pilot study explored the preliminary clinical signal of a combined non-invasive neuromodulation protocol with tDCS and tcVNS administered alongside supportive psychological counseling in a clinical sample of 12 adults with anxiety and depressive symptomatology.

The results indicated statistically significant and clinically meaningful reductions in both outcome measures, with large-magnitude effect sizes and consistent improvement across the entire sample (T = 0 on the Wilcoxon test for both BAI and BDI), and the proportion of participants achieving minimal symptom range on the BDI increased from 8.3% to 83.3%, and the prevalence of severe anxiety (BAI) decreased from 83.3% to 8.3%, over just 10 intervention sessions.

### 4.2. Hypothesized Neurobiological Mechanisms

The interpretations offered below are necessarily speculative: given the single-arm design, the absence of physiological markers of target engagement (e.g., heart rate variability, quantitative EEG), and the concurrent psychological counseling component whose independent contribution cannot be separated from neuromodulation effects, all mechanistic accounts must be treated strictly as working hypotheses to be tested in controlled, biomarker-enriched trials. Observed outcomes reflect the multimodal package as a whole, as the independent contribution of the concurrent, unstandardized psychological counseling cannot be separated from neuromodulation effects. The observed reductions are interpretable through the complementary neurobiological mechanisms of the two techniques, as tDCS modulates cortical excitability by shifting neuronal membrane potential: anodal stimulation increases excitability in the left DLPFC, a region that exerts top-down inhibitory control over the amygdala and supports adaptive emotional processing while cathodal stimulation of the right hemisphere concurrently reduces hyperactivity associated with depressive rumination. Together, these effects restore the frontal alpha asymmetry that has been linked to negative mood states [23,34,35]. tcVNS activates afferent vagal fibers at the cervical level that project to the nucleus tractus solitarius (NTS), with ascending connectivity to the locus coeruleus (noradrenaline), the dorsal raphe nucleus (serotonin), and the limbic system [16,17]. Increased vagal tone reduces sympathetic activation, an effect directly documented for the cervical approach, where tcVNS attenuated sympathetic reactivity to acute stress in a clinical sample [20]. Vagal stimulation also decreases inflammatory markers (IL-6, TNF-α) associated with depression and anxiety, and potentiates neuroplasticity through BDNF release [18,19].

Whether the sequential application of both techniques within the same session produces synergistic effects, with tDCS preparing the cortical substrate for enhanced receptivity to vagal modulation and vice versa, remains an open empirical question that the present single-arm design cannot resolve. Neuroimaging evidence motivating this hypothesis comes from Sun et al. [36], who found that simultaneous tDCS and auricular taVNS together produced greater brain activation than the numerical sum of either technique applied alone, particularly in the thalamus, pallidum, parahippocampal gyrus, dorsal raphe nucleus, substantia nigra, and periaqueductal gray, circuits central to mood regulation and monoaminergic tone. This potentiation was subsequently replicated at the behavioral level for working memory performance [37]. Critically, however, Sun et al. used simultaneous delivery and the auricular VNS variant, whereas the present protocol employed sequential administration and cervical stimulation; whether a supra-additive interaction generalizes across these differences is an empirical question the factorial trial described in Section 4.5 is designed to answer.

The within-group effect sizes observed here (r = 0.883; d ≈ −1.67 for anxiety, d ≈ −2.11 for depression) markedly exceed the magnitudes reported by meta-analyses of tDCS monotherapy for depression. In one of the most comprehensive syntheses to date (23 RCTs, N = 1092), authors estimated an active-versus-sham effect of g = 0.46 (95% CI [0.22, 0.70]), with response and remission rates of 33.3% and 19.1%, respectively, versus 16.6% and 9.8% in sham conditions [38]. Another study reported g = 0.37, consistent with Meron et al. (2015), and an individual participant data meta-analysis confirmed a similarly modest effect (β = 0.31; response 30.9% vs. 18.9%; remission 19.9% vs. 11.7%) [39,40,41]. The combined protocol evaluated here thus generated effects substantially larger than those consistently attributable to tDCS applied in isolation.

For anxiety, where the evidence for tDCS remains predominantly mechanistic, one study showed that prefrontal stimulation reduces amygdala reactivity to threat; the clinical magnitude observed in the present sample exceeds what is reported by available monotherapy studies, supporting the hypothesis that the combination of the two techniques leverages complementary mechanisms [42].

The conceptual backdrop for this expectation is the SELECT-TDCS factorial trial, in which combining tDCS with sertraline yielded response and remission rates (63.3% and 46.7%) superior to both tDCS monotherapy (43.3%/40.0%) and placebo (16.7%/13.3%) [43]. Crucially, SELECT-TDCS was able to make that inference because its factorial design formally estimated the tDCS × sertraline interaction term, a statistical capability the present single-arm pilot entirely lacks. The argument that a second mechanistically distinct component amplifies the clinical response therefore cannot be applied retrospectively to the present results; it instead motivates the adequately powered factorial trial proposed in Section 4.5, in which separate arms for tDCS alone, tcVNS alone, and their combination would allow interaction effects to be formally estimated.

The remission rates recorded here (BDI: 8.3%→83.3%) are numerically in the range reported by the combined arm of SELECT-TDCS [43]. However, this descriptive parallel cannot serve as evidence of synergy, as the present study lacks the factorial structure needed to attribute outcomes to the combination rather than to each component.

Controlled evidence for the superiority of combined tDCS + vagal stimulation over monotherapy has begun to emerge in neurological populations. A study demonstrated that the tDCS + taVNS combination produced significantly greater improvements in gait, balance, and daily functioning than either technique alone [44]. Similarly, the study of Liu et al. [45] registered a three-arm RCT protocol formally testing tDCS, taVNS, and their combination in post-stroke cognitive impairment, and Zhang et al. [46] extended this multimodal logic to a TMS + taVNS combination in mild cognitive impairment. Together, these initiatives mark a broader transition toward systematic clinical testing of combined neuromodulation strategies.

The reductions of 52.0% in BAI and 55.9% in BDI exceed the average reductions of 30–40% reported in tDCS protocols of comparable intensity applied over 10 sessions [24,43]. This difference of approximately 15–25 percentage points relative to historical monotherapy benchmarks is descriptively compatible with an additive or synergistic contribution from the cervical vagal stimulation component but, without a parallel sham or monotherapy arm, spontaneous recovery, regression to the mean, and non-specific treatment effects cannot be excluded. Regarding the specific cervical approach used here, most clinical tVNS studies for affective disorders have focused on the auricular branch, where meta-analyses confirm significant antidepressant effects relative to sham [21,22]. The cervical approach, which targets the vagal trunk directly, has documented effects on autonomic regulation and stress reactivity, and its application within a combined tDCS framework in affective pathology represents a clinically novel configuration [20].

Whether the magnitude of clinical effects observed in the present sample reflects a genuine synergistic interaction between tDCS and tcVNS of the kind suggested by the supra-additive brain activation documented by Sun et al. for the simultaneous auricular variant cannot be determined from an uncontrolled single-arm design. Extending this test to the cervical approach under sequential administration conditions, in a factorial design capable of estimating an interaction term, is a primary motivation for the trial proposed in Section 4.5 [36,47,48].

The exploratory stratification and multivariable continuous modeling converge to suggest preliminary, hypothesis-generating patterns regarding baseline severity and age. This severity-dependency trend aligns with the prior literature indicating that elevated baseline symptom burden often leaves wider latitude for measurable absolute improvement [49,50,51]. Whereas independent tDCS trials frequently report diminished efficacy in older adults due to age-related prefrontal atrophy [49,50], our descriptive observations revealed marked gains among older participants, and baseline age contributed positively within the multivariable regression.

A plausible, albeit speculative, neurobiological hypothesis is that concurrent tcVNS provides an auxiliary bottom-up autonomic pathway that compensates for cortical limitations, potentially addressing age-related reductions in baseline vagal tone.

However, because no concurrent physiological indices (such as heart rate variability or quantitative EEG) were recorded to confirm vagal or cortical target engagement, and because these descriptive patterns reflect a restricted subsample, these mechanistic interpretations must be treated strictly as hypotheses to be tested in prospective, biomarker-controlled trials rather than established outcomes.

### 4.3. Practical Implications

The transition of the majority of participants from the severe category to lower severity or remission over just 10 sessions suggests that the combined tDCS + tcVNS protocol warrants investigation as a complementary therapeutic option, particularly for patients who do not respond optimally to pharmacotherapy or conventional psychotherapy, or who present contraindications to these.

Pending confirmation in larger controlled trials, these preliminary findings raise the hypothesis that combined neuromodulation could eventually assist clinicians in identifying candidate patient profiles—specifically older individuals presenting with comorbid anxiety and depression and higher baseline symptom burden. If replicated in confirmatory studies, the rapid remission observed over 10 sessions could also suggest potential healthcare efficiency and long-term benefits for clinical settings. Furthermore, the exploratory associations—such as the correlation between age and initial depression severity (ρ = 0.60, p_uncorrected_ = 0.040) and between post-intervention anxiety and depression reduction (ρ = 0.73, p_uncorrected_ = 0.007)—generate preliminary hypotheses that older patients may require targeted depressive monitoring and that concurrent anxiety management may be key to optimizing antidepressant responses.

### 4.4. Strengths and Limitations

A primary strength of this study is its clinical novelty: by combining cervical vagal stimulation with tDCS in adults with anxiety and depression, it introduces a mechanistically distinct dual-modality configuration. The protocol leverages the complementary top-down (cortical) and bottom-up (autonomic) pathways of the two techniques while building on the independently established efficacy of each component [20,21,22]. Both interventions are non-invasive, portable and safe, suggesting potential feasibility for future clinical implementation, pending confirmation in controlled trials.

Several limitations must be acknowledged. First, the small sample (n = 12) and the absence of a control group preclude causal attribution of the observed effects to the intervention specifically, as regression to the mean, placebo effects, and therapeutic attention cannot be excluded. This is particularly salient given the uniform T = 0 finding across all participants, which is heavily influenced by positive participant expectancy, the non-specific therapeutic alliance resulting from ten consecutive daily clinical contacts, and statistical regression to the mean in patients presenting with severe baseline anxiety (BAI ≥ 26). The absence of formal DSM-5 or ICD-11 diagnostic confirmation via structured clinical interview limits the clinical interpretability of the findings and the generalizability of the results to defined diagnostic categories. Second, supportive psychological counseling was delivered concurrently with neuromodulation and was unstandardized across participants. This lack of standardization compromises internal validity and renders the intervention components non-isolable, meaning that the observed clinical improvements cannot be attributed to neuromodulation alone but rather reflect the combined effect of the multimodal therapeutic package. Third, the absence of randomization and an active sham-comparison group, essential for formally testing the synergy hypothesis, reduces inferential power. Fourth, no follow-up assessment was conducted; the durability of observed symptom reductions therefore cannot be determined. Future trials should include assessments at minimum at one and three months post-intervention. Fifth, sampling bias and individual neurobiological heterogeneity represent two distinct, compounding limitations that must be explicitly separated. On the one hand, reliance on a single-center, self-selected pilot cohort (n = 12) introduces a population diversity deficit that inherently restricts external validity. On the other hand, treating participants who present with comparable psychometric symptom severity (BAI/BDI) as biologically equivalent overlooks individual neurobiological and clinical heterogeneity (e.g., the wide age span of 22–75 years, associated variability in neuroplasticity, baseline autonomic/vagal tone, unrecorded psychiatric comorbidities, and heterogeneous treatment histories), which compounds sampling limitations rather than substituting for them [52]. In the absence of direct physiological tracking (HRV, qEEG), these compounding sources of variance preclude mechanistic conclusions. Sixth, the subgroup responder threshold was data-driven and exploratory, requiring validation in larger cohorts, and continuous multivariable modeling confirmed that baseline parameters operated as preliminary non-significant predictive trends (*p* > 0.05) rather than established clinical prognostic markers. Seventh, the substantial gender imbalance (83.3% female, n = 10 females vs. n = 2 males) limited statistical power for gender-based moderator analyses. Although a study demonstrated that the association between emotional intelligence, anxiety, and depression is not moderated by gender, gender differences in HPA axis reactivity and hormonal modulation of mood may nonetheless have influenced baseline symptom profiles and treatment trajectories in ways that cannot be evaluated within the present design [53]. Eighth, bivariate correlations were evaluated without family-wise multiplicity correction; although omitting derived percentage metrics restricted the matrix from 45 to 28 pairwise comparisons and eliminated circularity, the risk of Type I error remains elevated, requiring unadjusted correlation coefficients to be interpreted as strictly exploratory. Furthermore, the cervical neuromodulation component employed a PEMF-based device rather than conventional electrical tcVNS, and while both approaches target the cervical vagal region non-invasively, the underlying physical mechanisms differ, and findings from the electrical tcVNS literature cannot be assumed to transfer directly to PEMF-based cervical stimulation.

The non-invasive and portable nature of both components further supports feasibility for integration into routine clinical settings, particularly for patients who do not respond optimally to pharmacotherapy or who present contraindications to conventional interventions, pending confirmation in adequately powered controlled trials.

### 4.5. Future Directions

The results of this proof-of-concept pilot study justify proceeding to a more rigorous experimental design, consistent with the staged research pipeline advocated for feasibility pilot studies in neuromodulation. A randomized, sham-controlled, factorial trial with arms for tDCS alone, tcVNS alone, tDCS + tcVNS combined, and dual sham would allow formal estimation of the interaction term and unambiguous causal attribution.

Investigation of differential effects through selective deactivation paradigms would further clarify the relative contribution of each component. Integration of physiological biomarkers (HRV as a marker of vagal tone; quantitative EEG as an index of cortical excitability changes) would elucidate mechanistic pathways. Evaluation of booster or maintenance sessions could inform protocols for sustaining treatment gains over time.

## 5. Conclusions

This proof-of-concept feasibility pilot study demonstrated that a multimodal intervention package integrating tDCS, tcVNS, and concurrent supportive psychological counseling was associated with statistically significant and clinically meaningful reductions in anxiety and depressive symptomatology in all 12 included participants. The large-magnitude and entirely consistent effects justify further testing of this dual non-invasive neuromodulation approach in rigorous experimental frameworks. Because the present pre-experimental design does not permit separation of specific intervention effects from non-specific factors, and because no published trial is yet to formally examine the tDCS + tcVNS interaction in mood and anxiety disorders, the conclusions reported here remain inherently preliminary. Exploratory observations suggested descriptive associations between older age, greater baseline severity, and larger symptom reductions; these are hypothesis-generating only and do not constitute established predictors of response. Adequately powered, randomized, sham-controlled, and preferably factorial trials are required to determine whether the observed symptom reductions following the complete intervention package reflect genuine therapeutic efficacy beyond non-specific effects, as the present uncontrolled design cannot establish comparative efficacy or synergy.

## Figures and Tables

**Figure 1:**
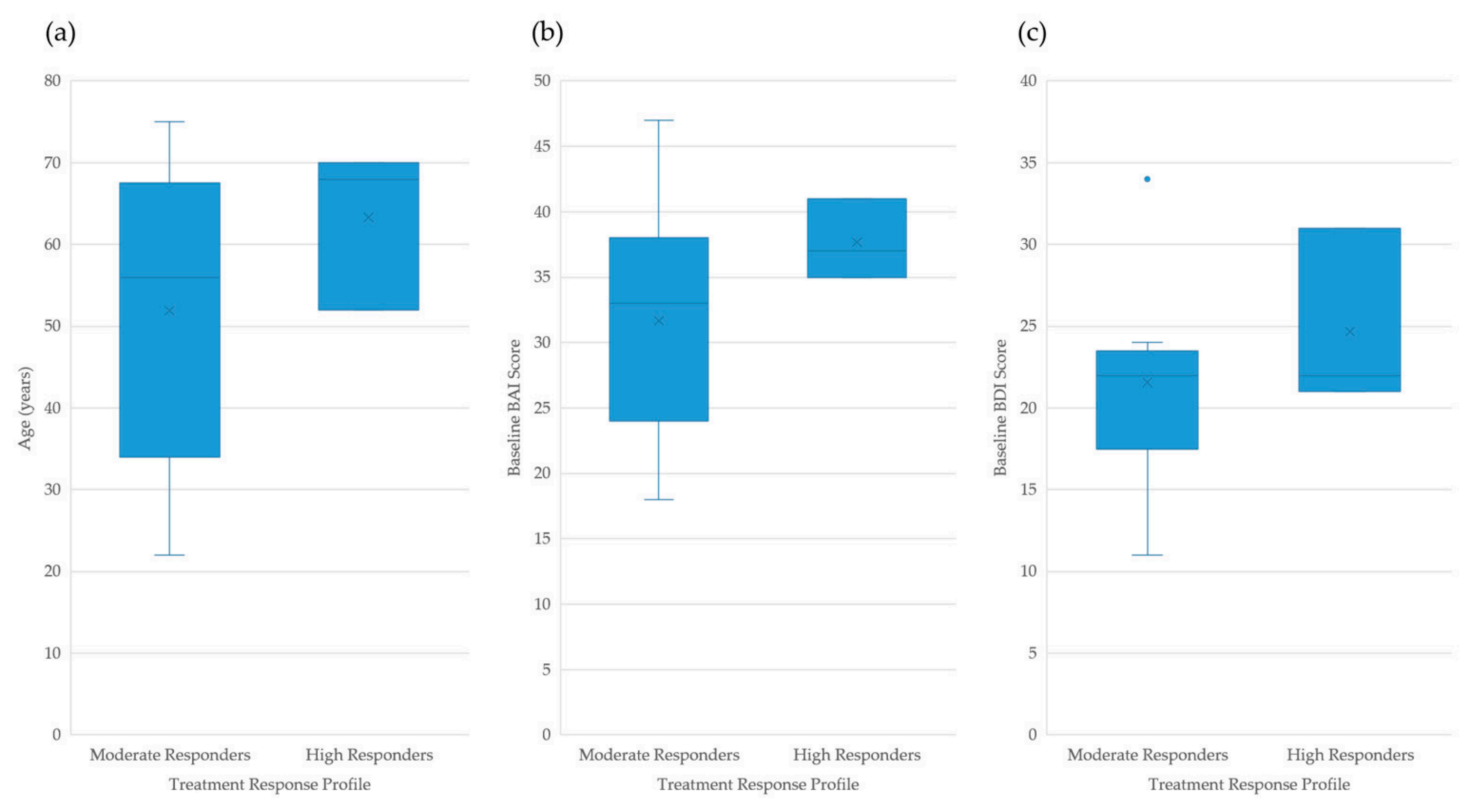
Baseline demographic and clinical characteristics stratified by treatment response profile: (**a**) age distribution; (**b**) baseline Beck Anxiety Inventory (BAI) scores; (**c**) baseline Beck Depression Inventory (BDI) scores. In each boxplot, the horizontal line within the box represents the median, the cross (×) denotes the group mean, and the lower and upper box boundaries correspond to the 25th (Q_1_) and 75th (Q_3_) percentiles (interquartile range, IQR). Whiskers extend to the minimum and maximum observed values within 1.5 × IQR; the individual circular point in panel (**c**) indicates a statistical outlier belonging to the Moderate Responder group (participant P09, baseline BDI score of 34). Sample size (n = 12): Moderate Responders (n = 9); High Responders (n = 3).

**Table 1:** Individual BAI and BDI scores pre- and post-intervention ^1^.

Patient	Age (Years)	BAI Pre	BAI Post	BDI Pre	BDI Post	ΔTotal
P01	52	41	3	21	0	↓59
P02	56	38	24	22	13	↓23
P03	29	47	35	15	11	↓16
P04	22	26	19	23	9	↓21
P05	45	37	23	22	10	↓26
P06	68	35	3	31	9	↓54
P07	39	38	23	11	6	↓20
P08	70	37	6	22	2	↓51
P09	75	33	19	34	21	↓27
P10	67	18	9	24	15	↓18
P11	68	22	14	23	11	↓20
P12	66	26	13	20	11	↓22
M (SD)	54.75 (17.47)	33.17 (8.47)	15.92 (9.73)	22.33 (6.07)	9.83 (5.56)	29.75 (15.53)
Median	61.0	36.0	16.5	22.0	10.5	22.50
Range	22–75	18–47	3–35	11–34	0–21	16–59

^1^ n = 12. M = mean; SD = standard deviation; Δtotal = sum of pre–post differences across BAI and BDI; ↓ decreasing value.

**Table 2:** Shapiro–Wilk normality tests ^1^.

Variable	Mean (SD)	95% CI	Skewness (SE)	Kurtosis (SE)	W	*p*
BAI–pre	33.17 (8.47)	[27.78, 38.55]	−0.385 (0.637)	−0.497 (1.232)	0.947	0.593
BAI–post	15.92 (9.73)	[9.74, 22.10]	0.308 (0.637)	−0.341 (1.232)	0.948	0.613
ΔBAI	−17.25 (10.35)	[−23.83, −10.67]	−1.179 (0.637)	−0.011 (1.232)	0.799	0.009
BDI–pre	22.33 (6.07)	[18.48, 26.19]	0.185 (0.637)	1.043 (1.232)	0.917	0.260
BDI–post	9.83 (5.56)	[6.30, 13.36]	0.050 (0.637)	0.875 (1.232)	0.953	0.685
ΔBDI	−12.50 (5.93)	[−16.27, −8.73]	−0.383 (0.637)	−0.804 (1.232)	0.925	0.329

^1^ Δ = post-minus-pre-difference score (negative values signify clinical symptom reduction). SD = standard deviation; CI = confidence interval; SE = standard error (0.637 for skewness, 1.232 for kurtosis across all indices, derived from sample size n = 12); W = Shapiro–Wilk test statistic; *p* = two-tailed significance.

**Table 3:** Wilcoxon signed-rank test: pre–post comparisons and paired confidence intervals (n = 12) ^1^.

Variable	M Pre (SD)	M Post (SD)	Mean Change [95% CI]	T	Z	*p*	r
BAI	33.17 (8.47)	15.92 (9.73)	−17.25 [−23.83, −10.67]	0	−3.059	<0.001	0.883
BDI	22.33 (6.07)	9.83 (5.56)	−12.50 [−16.27, −8.73]	0	−3.059	<0.001	0.883

^1^ M = mean; SD = standard deviation; CI = confidence interval; T = Wilcoxon statistic; Z = standardized test statistic (p_exact_ < 0.001, p_asymp_ = 0.002); r = effect size.

**Table 4:** Effect sizes and clinical relevance ^1^.

Variable	M Pre	M Post	ΔM	% Reduction	Cohen’s d	r Wilcoxon
BAI	33.17	15.92	−17.25	52.0%	−1.667	0.883
BDI	22.33	9.83	−12.50	55.9%	−2.107	0.883

^1^ M = mean; ΔM = mean pre–post difference; d = Cohen’s d on paired differences; r = Wilcoxon r effect size.

**Table 5:** Distribution of patients by clinical severity category, pre- and post-intervention ^1^.

	BAI (Anxiety)		BDI (Depression)	
Clinical category	Pre N (%)	Post N (%)	Pre N (%)	Post N (%)
Minimal symptom range (0–13)	0 (0.0%)	3 (25.0%)	1 (8.3%)	10 (83.3%)
Mild	0 (0.0%)	3 (25.0%)	1 (8.3%)	1 (8.3%)
Moderate	2 (16.7%)	5 (41.7%)	8 (66.7%)	1 (8.3%)
Severe	10 (83.3%)	1 (8.3%)	2 (16.7%)	0 (0.0%)

^1^ BAI thresholds: 0–7 minimal, 8–15 mild, 16–25 moderate, ≥26 severe. BDI thresholds: 0–13 minimal symptom range, 14–19 mild, 20–28 moderate, ≥29 severe [25].

**Table 6:** Spearman rank-order correlation matrix ^1^.

Variable	1	2	3	4	5	6	7	8
1. Age	—							
2. BAI pre	−0.41	—						
3. BAI post	−0.52	0.34	—					
4. BDI pre	0.60 *	−0.63 *	−0.36	—				
5. BDI post	0.28	−0.42	0.39	0.41	—			
6. ΔBAI	−0.25	−0.58 *	0.40	0.16	0.55	—		
7. ΔBDI	−0.40	0.13	0.73 **	−0.54	0.46	0.44	—	
8. ΔTotal	0.41	0.21	−0.56	0.25	−0.43	−0.77 **	−0.82 **	—

^1^ n = 12. ΔBAI and ΔBDI represent post-minus-pre-raw differences; ΔTotal represents cumulative absolute symptom reduction across both scales; ** *p* < 0.01; * *p* < 0.05 (two-tailed unadjusted values reported for exploratory heuristic purposes).

**Table 7:** Baseline characteristics and clinical outcomes stratified by treatment response profile ^1^.

Variable	Moderate Responders (n = 9)	High Responders (n = 3)			Total Sample (n = 12)
		P01	P06	P08	
**Age (years)**					
M (SD)/Individual scores	51.89 (18.94)	52	68	70	54.75 (17.47)
Mdn [Min, Max]	56.00 [22,75]	68.00			61.00 [22,75]
**Baseline BAI**					
M (SD)/Individual scores	31.67 (9.29)	41	35	37	33.17 (8.47)
Mdn [Min, Max]	33.00 [18,47]	37.00			36.00 [18,47]
**Baseline BDI**					
M (SD)/Individual scores	21.56 (6.35)	21	31	22	22.33 (6.07)
Mdn [Min, Max]	22.00 [11,34]	22.00			22.00 [11,34]
** ΔTotal (points**)					
M (SD)/Individual scores	21.44 (3.57)	59	54	51	29.75 (15.53)
Mdn [Min, Max]	21.00 [16,27]	54.00			22.50 [16,59]

^1^ n = 12. M = mean; SD = standard deviation; Mdn = median; Min = minimum; Max = maximum.

**Table 8:** Exploratory multivariable linear regression model evaluating baseline continuous covariates on cumulative symptom reduction (ΔTotal, n = 12) ^1^.

Variable	B	SE	β	t	*p*	95% CI for B
(Constant)	−36.510	29.779	—	−1.226	0.255	[−105.18, 32.16]
Baseline BAI Score	0.997	0.544	0.548	1.834	0.104	[−0.26, 2.25]
Age (years)	0.313	0.313	0.355	1.000	0.347	[−0.41, 1.04]
Baseline BDI Score	0.718	0.887	0.282	0.810	0.441	[−1.33, 2.76]

^1^ Dependent variable = cumulative absolute score drop across inventories (ΔTotal). B = unstandardized regression coefficient; SE = standard error; β = standardized regression coefficient; t = Student’s t statistic; *p* = two-tailed significance; CI = confidence interval.

**Table 9:** Non-parametric Mann–Whitney U test comparing clinical improvement across genders ^1^.

Outcome Measure	Male (n = 2) Mean Rank	Female (n = 10) Mean Rank	Mann–Whitney U	Wilcoxon W	Z	Asymp. *p*	Exact *p*
%BAI Reduction	8.50	6.10	6.000	61.000	−0.861	0.389	0.485
%BDI Reduction	8.00	6.20	7.000	62.000	−0.645	0.519	0.606
ΔTotal	7.25	6.35	8.500	63.500	−0.323	0.747	0.758

^1^ n = 12 (n = 2 males, n = 10 females). U = Mann–Whitney test statistic; W = Wilcoxon rank-sum statistic; Z = standardized test statistic; Asymp. *p* = two-tailed asymptotic significance; Exact *p* = two-tailed exact significance calculated for small sample size and tied ranks. %BAI Reduction = percentage score decreases from baseline on the Beck Anxiety Inventory; %BDI Reduction = percentage score decreases from baseline on the Beck Depression Inventory. Statistical significance threshold set at *p* < 0.05.

## Data Availability

The data presented in this study are not publicly available due to privacy and ethical restrictions concerning the protection of participants’ personal and clinical information. The data may be made available from the main author upon reasonable request and subject to appropriate ethical and privacy considerations.

## Abbreviations

The following abbreviations are used in this manuscript:

tDCS	Transcranial Direct Current Stimulation
tcVNS	Transcutaneous Cervical Vagus Nerve Stimulation
BAI	Beck Anxiety Inventory
BDI	Beck Depression Inventory
DLPFC	Dorsolateral Prefrontal Cortex
SSRI	Selective Serotonin Reuptake Inhibitor
HRV	Heart Rate Variability
BDNF	Brain-Derived Neurotrophic Factor
taVNS	Transcutaneous Auricular Vagus Nerve Stimulation
TES	Transcranial Electrical Stimulation
PEMF	Pulsed Electromagnetic Field
qEEG	Quantitative Electroencephalography
NTS	Nucleus Tractus Solitarius
RCT	Randomized Controlled Trial
SD	Standard Deviation
Mdn	Median
IQR	Interquartile Range
CI	Confidence Interval
SE	Standard Error
